# Medical Opinion and Movement

**Published:** 1910-04-02

**Authors:** 


### Medical Opinion and Movement.

### Pathology of Gout.

At a meeting of a Society of Medicine in Berlin Dr. Brugsch gave an interesting account of his researches into the pathology of gout. He finds that gout is determined by a disorder of the nucleinic metabolism. Analysis of the blood shows that there is no retention of uric acid in the gouty. In normal individuals, as well as in the gouty and arthritic, after injection of nucleinic substances only half is found in the urine in the form of uric acid, the rest being transformed into urea by the uricolytic ferment. The same thing happens if uric acid is injected in solution. There is, therefore, no deficiency of uricolysis in the gouty. The author distinguishes three diastases which co-operate in the production of uric acid from the nucleins. The nucleins are first converted into aminopurines by nuclease, and these are then decomposed into oxypurines by disamidase, and finally xanthinoxydase oxidises the oxypurines into uric acid. The author fixes upon the disamidase as the chief deficient factor in the gouty condition. He finds that aminopurines such as adenine, eliminated in a normal person within two days in the form of uric acid and urea, take in the gouty four or five days in the process of elimination. There is probably also a deficiency in nuclease, as after injection of nucleins six or seven days are required for their elimination in the gouty condition. On the other hand, the author admits that certain rare cases do occur in which there is deficient uricolysis, and he reports a case in which all the nuclein appears in the urine in the form of uric acid, and consequently any injection of nucleinic substances results in the accumulation of uric acid in the blood.

### Mountain Sickness.

At a meeting of the Medical Society of Berlin Dr. Knoche gave an account of his experiences and observations in regard to mountain sickness during his sojourn among the heights of the Cordilleras of Peru. There was a steady increase in the number of red corpuscles, so that at the end of three months they amounted to 7½ millions in himself and 7 millions in his wife. This increase, however, was not permanent, but gradually fell off till at the end of six months the number was actually below the normal—*i.e.* 4,500,000. The same conditions prevailed with the servant, who was a native. On the other hand, the hæmoglobin-content of the blood remained stationary at 120 per cent. He suggests that the ideas acquired from Alpine experiences in regard to the pathology of mountain sickness are very imperfect. In Peru and Bolivia attacks known as "*houna*" or "*sirroche*" are ascribed by the natives to certain orographic or topographic conditions other than simple altitude. Mule-drivers avoid certain paths because they are "haunted" by *houna*, and again certain valleys are known for the frequent occurrence of *sirroche*. In one such valley the whole of the author's caravan was affected by the complaint. In La Paz, the capital of Bolivia, certain streets are known for the number of cases of this malady which occur there. *Sirroche* consists in a condition of nervous excitation. It is not due to a deficiency of oxygen, as it may occur after a descent of 5,000 metres. It does not appear to depend upon muscular fatigue, as it is especially liable to appear in the night and disappear during the day. It may be accompanied by hæmorrhages, which may attain to a dangerous degree. The author makes the interesting suggestion that the cause of this affection may be of an electric nature, such as electric discharges in the air. In support of this hypothesis he points out that the conditions favouring the accumulation of electricity are just those which predispose to the occurrence of this affection: mines and veins of metal in the hills, closed valleys, mountain peaks liable to attract and discharge atmospheric electricity, etc. On the other hand, he does not deny that the rarity of the atmosphere, and consequent deficiency of oxygen, may be a predisposing cause.

### Tubercular Infection from Milk.

An extensive inquiry has been carried out in Germany during the years 1905-9 under Government auspices to investigate this question of possible infectivity of human individuals through milk from tuberculous cows, and the report by Dr. A. Weber has just appeared. In every case of tuberculous udders of a cow ascertained by the official veterinary, an inquiry was instituted in regard to the consumers of the milk from that cow, whether it had been consumed unmixed with milk from other cows, and in the fresh state or boiled, how long it had been consumed, and whether there were any cases of tuberculosis infection among the consumers. Altogether there were 69 cases in which it was definitely ascertained that milk had been consumed in a fresh state or in the form of butter, cheese, etc., for a varying length of time, and these 69 cases involved 360 people, including 151 children. Apart from a few very doubtful cases, there were only two cases in which consumption of this fresh tuberculous milk was followed by a development of the disease. Both cases were very young children, and the affection was one of the cervical glands, the condition was of a mild nature, and the general health was not affected. In both cases it was the youngest of the family affected, and the rest of the family, several in number, all of whom had taken the same milk, remained healthy. On the other hand, it is stated that in both cases the affection of the udders was of a severe nature, and the milk was only mixed with that of one other cow. In the one case the child had the milk for a year and a half, and in the other for a year. It would appear, therefore, from this extensive inquiry that infection through milk is by no means common or easy, that, in fact, to produce the disease prolonged feeding with badly-infected milk is necessary, and that even then the disease is of a mild nature.

**Radial Emanations.**

The therapeutic value of radium emanations is still vague and uncertain. We have still to ascertain the manner in which the emanations act within the organism. With this idea in view Drs. Kohlrausch and Plato have conducted some experiments to study the effects of the ingestion of liquids charged with emanations of radium and of prolonged immersion in baths similarly charged. They employed special sensitive apparatus capable of measuring infinitesimal quantities of radium. It has been much debated whether under such conditions the urine becomes radioactive. According to their results the answer is in the negative. Both after ingestion of the fluid and also after prolonged immersion in the bath they have failed to find any evidence of radium in the urine. Another fact which their researches disclose is that the skin is impermeable to the radium emanations. After immersion in the bath the radium emanations do not appear to penetrate within the organism except through the lungs, and according to these authors it is also by the lungs that these emanations also leave the system after injection of the radium fluid.

**Prophylaxis of Peritonitis.**

The subject of post-operative peritonitis and the improvement of prophylactic measures for its prevention has been investigated by Dr. Wilkie, whose experimental and other research is published in the *Medical Chronicle*. It is especially after resection of intestine and similar operations that contamination of the peritoneum is still not infrequent; and the author gives the operative mortality of intestinal resection as still about 20 per cent. In these infections the bacillus coli communis plays a leading rôle, and it is to the fortification of the peritoneum against its ravages that Dr. Wilkie has devoted most attention. Recovery, he says, depends on the patient's capacity, and particularly on the capacity of his peritoneum, to react promptly. The presence of a leucocytosis in the blood-stream favours such prompt peritoneal reaction. In animals the preliminary subcutaneous injection of a suitable vaccine, together with nucleic acid, enhances the powers of recovery; and this combination is of much greater value than is either agent alone. A vaccine made from a virulent culture is more efficacious than one made from an old stock culture of the same germ. Further, the conclusion is drawn that two-stage operations for the removal of intestinal growths have, amongst other advantages, that of causing an auto-vaccination at the first operation which enables the peritoneum more easily to withstand infection should it occur at the second one.

**The Thalamic Syndrome.**

The original and classical description of this morbid entity we owe to Déjerine, whose first published account of it is now six years old; in all some 18 cases have been reported, most of which conform closely to Déjerine's account. The latest of these is reported by Dr. Jelliffe in the *Medical Record*. Shortly, the patient was a man of 40 with a syphilitic infection of 13 years' standing, adequately treated (apparently) at the time. He went on well after this infection until he had an apoplectiform attack of a not very severe sort, from which in a few weeks he completely recovered. A year later he had a second attack, but without loss of consciousness, and a month later still he was found to exhibit: (1) slight hemiparesis of the right upper and lower extremities; (2) marked ataxia in the right arm, slight in the right leg; (3) choreoathetoid movements of the right arm; (4) disturbance of pain sense, of deep sensibility, and of postural sense in the arm, with very slight corresponding changes in the leg; (5) astereognosis in the right hand; (6) and definite, but not marked, pains in the right shoulder. Clinically this is a good example of the thalamic syndrome of Déjerine; the only important variation from the type is the absence of severe paroxysmal pains, and the author remarks that the shoulder may yet give trouble in this respect.

Autopsy on certain of the patients whose cases have been reported discloses the lesion which is responsible for this combination of symptoms. The primitive lesion occupies the posterior and external part of the optic thalamus at all its levels, but does not go beyond it above or below. Lower, the focus diminishes in extent, involves the external lateral nucleus, and above it disappears rapidly at the superior level of the thalamus. It destroys the posterior part of the posterior limit of the internal capsule and the posterior part of the lenticular nucleus. As for secondary degenerations, there are some which can be followed in the corona radiate to the base of the convolutions. Degeneration of the pyramidal tract is limited to a small portion just internal to Turck's bundle; it is less marked in the direct than in the crossed tract. The leumiscus is normal in the tegmentum of the peduncles, frons, and medulla. It follows, seemingly, that in the internal capsule the grouping of fibres as worked out by Horsley and Beevor in monkeys is not quite the same in man; for it was supposed that the anterior part of the posterior limit of the internal capsule corresponds to the upper, the posterior part to the lower extremity. A good bibliography of the cases of this curious syndrome to date is given.

**Tetanus from a Carious Tooth.**

In the same journal a case believed by the author, Dr. Luckett, to be unprecedented, is reported, wherein tetanus originated in the cavity of a carious tooth. The patient was aged 10 years, and had a bad habit of picking her teeth with straw, pins, or anything else that came handy; she was subject to toothache. When she came under observation she was suffering from tetanus, the symptoms of which had been developing gradually for four days. Examination failed to reveal any scratch, wound, or other portal of entry for the bacillus, nor was there any history of recent trauma. Some seven or eight days later death ensued, despite treatment by tetanus antitoxin, given by a spinal puncture. As the carious teeth appeared to be the only septic focus in the entire body, they were removed and submitted to a bacteriologist. Films made direct from the pus which bathed them showed streptococci, staphylococci, spirilla, diplococci, and other forms, but nothing identifiable as a tetanus bacillus was seen. A small part of one tooth was inserted subcutaneously into a guinea-pig, together with 0.5 c.c. of a saline suspension of the pus. A localised abscess followed, and for four days tetanic spasms occurred; the animal recovered. The pus from the abscess caused the death of two mice with spastic convulsions. Culture experiments were even more decisive, for the bacillus was actually isolated in pure culture by the method of Kitasato, and its pathogenicity proved by injections into mice and guinea-pigs. As the author justly remarks, there may be other cases of supposed idiopathic tetanus in which the point of entrance has been in the jaw, and his careful work shows the importance of dental sepsis and its prevention.

### X-Rays and Bismuth Subnitrate.

The serious and even fatal accidents which have at times followed the ingestion of large doses of bismuth subnitrate have been attributed to intoxication caused by the formation under certain conditions of nitrites in the system at the expense of the subnitrate. These nitrites are produced only in the presence of fæcal material, and, as Böhme has shown, the presence of intestinal micro-organisms is essential for their production. Seeing that practically all the serious cases of poisoning have occurred in patients who have been given large doses of bismuth subnitrate for radiographic purposes, Messrs. Marre and Taillander, as reported at a recent meeting of the Société de Biologie, have made experiments with the object of discovering whether the application of the $x$-rays counted for anything in the formation of these nitrites. As the result of their observations they have come to the conclusion that, so far from helping the formation of nitrites, the $x$-rays actually exert a definite restraining influence on the decomposition of the bismuth subnitrate. This result is in all probability effected by the action of the rays in hindering the activity of the intestinal flora.

### Periodic Hydrarthrosis.

At a meeting of the Société Médicale des Hôpitaux, Dr. Ribierre reported a case of periodic hydrarthrosis which he has had under his care for the past four years. The affection is characterised by the appearance at very regular intervals, and for many years, of hydrarthrosis, which usually occurs in one or both knees. These articular manifestations are painless, and are not accompanied by fever or redness of the joint. In the intervals between attacks the articulation seems perfectly normal. The condition for the most part attacks females at puberty and young adults, and may show spontaneous remissions. No satisfactory theory has as yet been evolved to explain these facts. In the case reported by the author no ætiology was discoverable. The patient was treated by thyroid medication, the drug being given in small doses over a very long period. The periodicity of the attacks was upset by this treatment, their intensity being also diminished. A rapid and complete amelioration of concomitant manifestations such as œdema of the integuments, psychic disturbances, migraine, etc., was noticed. Finally, the articular crises disappeared entirely, and for the last two years the recovery has been complete. The author entirely disagrees with those observers who advocate operative interference in these cases.

### A New Method of Diagnosing Syphilis.

A new method of diagnosing syphilis has been recently reported to the Société de Biologie by MM. Nicolas, Favre and Gautier. In this method a glycerinated extract of the liver of a fœtus suffering from congenital syphilis, and very rich in treponemata, is used, the resulting product being called by the authors syphiline. Up to the time of writing the cuti-reaction has given entirely negative results at the hands of the authors. The intra-dermo-reaction has, however, been successful in several cases. Twelve syphilitic patients were submitted to the experiment, ten being in the secondary and two in the tertiary stage of the disease. Seven very marked positive reactions were obtained in five of the secondary and the two tertiary cases. In four of the remaining cases the reaction was very feeble or doubtful, but it was negative in only one case. Three healthy patients who were submitted to the test as controls showed no reaction. The authors would welcome further study of their method with the object of discovering its true value and limitations.

### Familial Spleno-megaly.

In 1906 Schlagenhaufer described a type of enlargement of the liver and spleen with anæmia which is favourable in prognosis and familial in incidence. A. Plehn, in the *Deutsch. Med. Woch.*, reports three further cases. The disease is one which commences in early childhood and runs a long, chronic course. It is characterised by great enlargement of the spleen and liver and marked anæmia. Prognosis is favourable. The author's three cases occurred in one family, the father, son, and daughter being affected. The first case, that of the daughter, aged 18, showed anæmia and pallor, which had been present many years, a spleen occupying most of the left half of the abdomen, enlargement of the liver, and marked icterus. Blood examination revealed the presence of mast cells and nucleated red corpuscles, with a hæmoglobin index of 20 per cent. The father, a man of 61, who was next examined, had, as a child, suffered from spleno-megaly, anæmia, and jaundice, a condition which had, however, sufficiently improved with years to allow him to serve his time in the army, and to lead an active life. Examination showed that his spleen still reached six inches below the costal margin. The brother, aged 26, had had a large spleen and pallor from infancy, but had never been in ill-health. Examination showed that his spleen reached a hand's breadth below the costal margin, and he had 48 per cent. of hæmoglobin. In none of the cases could the condition be accounted for by any of the usual causes, such as malaria, syphilis, etc.

